# Relapse of Multiple Myeloma Presenting as Extramedullary Plasmacytomas in Multiple Organs

**DOI:** 10.1155/2015/452305

**Published:** 2015-01-28

**Authors:** Murat Köse, Ersida Buraniqi, Timur Selçuk Akpinar, Seyit Mehmet Kayacan, Tufan Tükek

**Affiliations:** Department of Internal Medicine, Istanbul Faculty of Medicine, Istanbul University, 34093 Istanbul, Turkey

## Abstract

Multiple myeloma is a neoplastic plasma cell disorder. It is characterized by collections of abnormal plasma cells accumulating in the bone marrow, where they interfere with the production of normal blood cells. It usually presents as a multisystemic involvement, whose symptoms and signs vary greatly. Some patients have slowly progressive disease while others have aggressive clinical behavior by extramedullary involvement. In addition to renal failure, anemia, hypercalcemia, lytic bone lesions, and immunodeficiency, it also affects multiple organ system, such as pancreas, adrenal glands, kidney, skin, lung, liver, spleen, lymph nodes, and bone. To raise awareness of the variable presentations of this disease, we report a 53-year-old male patient, with multiple myeloma in his first remission who relapsed with extramedullary plasmacytomas (EMPs) involving multiple organs, such as pancreas, adrenal glands, kidney, skin, lung, liver, spleen, and lymph nodes.

## 1. Introduction

Multiple myeloma (MM) is a neoplastic plasma cell disorder which usually presents as renal failure, anemia, hypercalcemia, lytic bone lesions, immunodeficiency, pathological fractures, and hyperviscosity. It constitutes 1% of all cancers and 10% of all hematological malignancies [1–3]. It usually occurs in the seventh or eighth decade of life [4]. Prognosis in MM is largely dependent on laboratory markers, such as *β*2 microglobulin, CRP, LDH, albumin, platelet count, and extramedullary involvement. Extraosseous involvement is rarely seen in MM. It is usually associated with advanced stage and exhibits aggressive behavior. Any organ or system can be affected. Involvement of solid organs in the abdominal region, mesentery, gastrointestinal tract, lung, pleura, nasal cavity, nasopharynx, parathyroid and thyroid glands, breast, testis, vagina, uterus, orbital cavity, meninx, kidney, stomach, muscle, and subcutaneous tissue has been reported so far [2, 4]. Particularly, involvement of adrenal glands and pancreas is exceedingly rare. To raise awareness of the variable presentations of this disease, we report a 53-year-old male patient, with multiple myeloma in his first remission who relapsed with extramedullary plasmacytomas (EMPs) involving multiple organs, such as pancreas, adrenal glands, kidney, skin, lung, liver, spleen, and lymph nodes.

## 2. Case Report

A 53-year-old male presented with back pain on April 2006. Laboratory test results revealed an elevated erythrocyte sedimentation rate of 151 mm/hour, anemia, gamma globulin at 2.24 g/dL, and M-spike 8.8%. Bone marrow aspiration and biopsy showed plasma cell infiltration with prominent monotypic pattern (kappa, with minimal lambda). Serum and urine electrophoresis exhibited monoclonal IgG kappa paraproteinemia. Clinical and laboratory findings confirmed the diagnosis of MM with Durie-Salmon stage 3A/ISS stage I.

**Figure 1:**
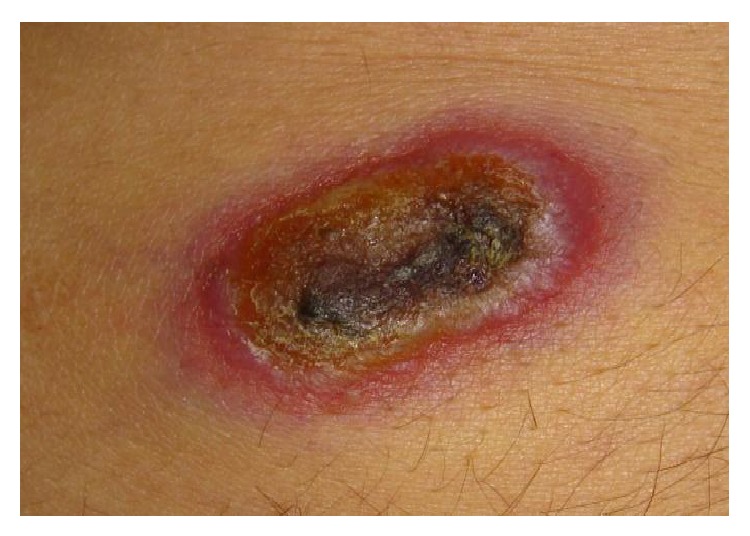
Crusted, raised skin lesion with regular borders.

The patient was treated with methylprednisolone, melphalan, and zoledronic acid. Complete response was obtained following six cycles. The patient was admitted to our clinic due to development of circumscribed, painless, red elevated lesions, fatigue, loss of appetite, jaundice of one-week duration, dark urine, pale stools, and itching, following remission lasting for 1 year. Past medical history was unremarkable and family history was noncontributory. Physical examination showed icteric sclera and skin, pale conjunctiva, crusted nodular lesions, measuring 2 × 3 cm in size (Figure 1), on upper extremities, axillary regions and upper right quadrant of abdomen, diffuse abdominal tenderness, and hepatomegaly (2 cm below right costal margin).

Complete blood count revealed leukocyte of 7,600/*μ*L, granulocyte 5,200/*μ*L, hemoglobin 14.1 g/dL, hematocrit 41.4%, and platelet 327.000/*μ*L. Other initial laboratory tests were as follows: blood urea nitrogen 13 mg/dL; serum creatinine 0.9 mg/dL; sodium 139 mmol/L; potassium K: 3.8 mmol/L; calcium 8.7 mg/dL; phosphorus 3.1 mg/dL; alkaline phosphatase 2223 U/L; aspartate transaminase 190 U/L; alanine transaminase 333 U/L; lactate dehydrogenase 612 U/L; total bilirubin 8.1 mg/dL; direct bilirubin 6.5 mg/dL; total protein 8.2 g/dL; albumin: 3.8 g/dL; erythrocyte sedimentation rate 61 mm/h. Serum protein electrophoresis revealed an M-spike of 2.14 g/dL in gamma globulin region. Urinalysis was insignificant except for bilirubinuria.

**Figure 2:**
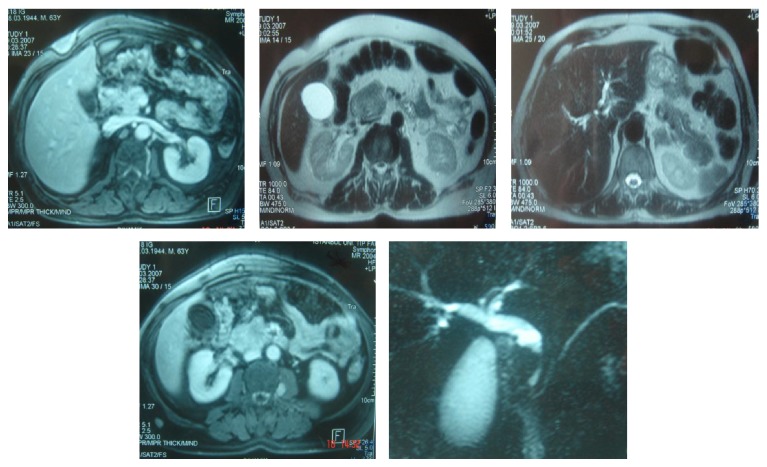
Multiple intra-abdominal lesions involving pancreas, adrenal glands. Common bile duct is dilated.

**Figure 3:**
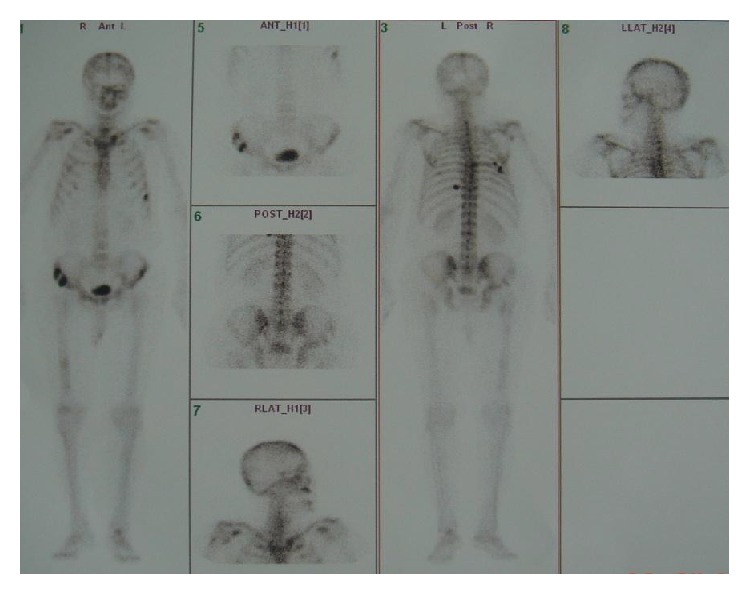
Bone scintigraphy, intense activity in multiple bones.

**Figure 4:**
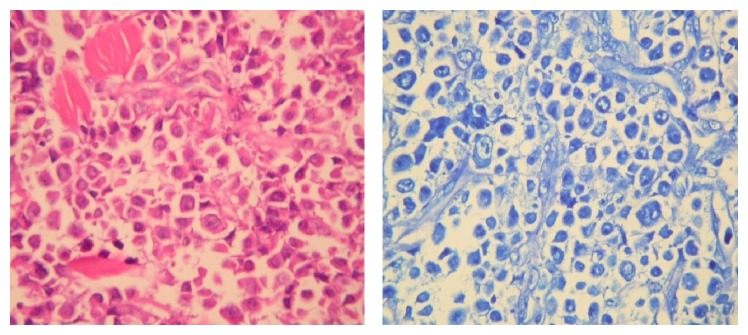
Skin biopsy samples show neoplastic cell infiltration and collagen bundles.

**Figure 5:**
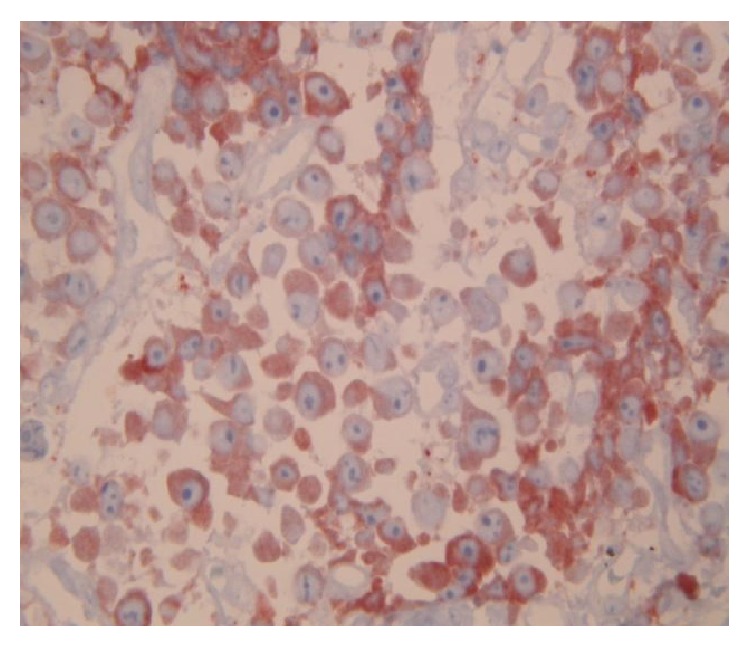
Skin biopsy and immunohistochemical staining reveals kappa monotypic plasma cell infiltration.

**Figure 6:**
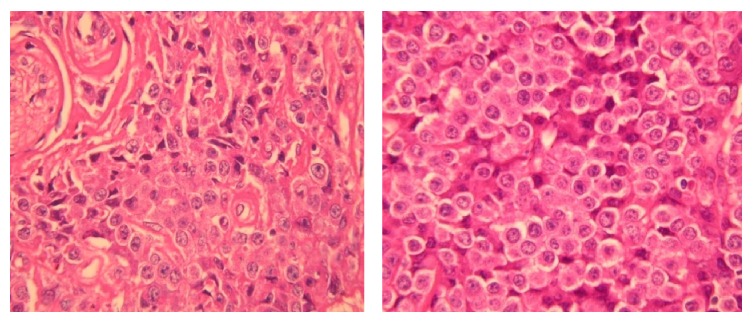
Biopsy of mass in the pancreatic head, neoplastic cell infiltration similar to skin biopsy findings.

**Figure 7:**
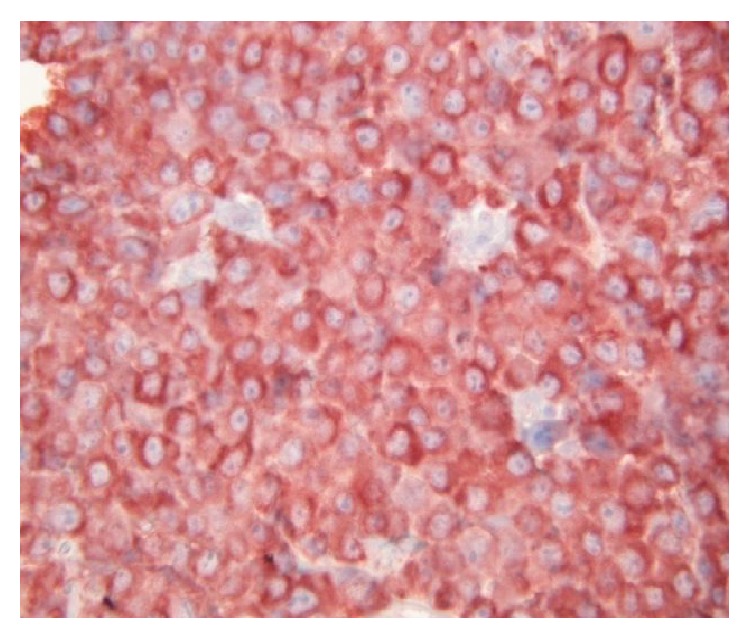
Biopsy of mass in the pancreatic head, kappa monotypic plasma cell infiltration.

Chest computed tomography showed a mass of 5 × 4 cm in size, destructing the rib of right chest wall, two nodular lesions of 2 cm and 3.5 cm in size, situated in subcutaneous fatty layer of right and left chest walls, respectively, and a seemingly benign lymph node of 1.2 cm in the perivascular space of mediastinum. Abdominal magnetic resonance imaging and MR cholangiopancreatography disclosed dilated intrahepatic biliary ducts, gall bladder hydrops with a 6 mm polyp, moderately dilated common bile duct (16 mm), a solid mass, 4.5 cm in diameter, in the pancreatic head, a regularly contoured mass measuring 26 × 18 mm in diameter in the left adrenal gland, a mass of 2 cm in the superior lobe of left kidney, and a mass of 2.8 cm in the inferior splenic pole; in addition, multiple masses varying in size were seen in the abdominal oblique muscle, left pararectal space, right iliac and ischial bones, sacroiliac wing, close proximity to the inferior pole of left kidney, and left perirectal fossa (Figure 2). Bone scintigraphy demonstrated increased activity in the anterolateral aspect of eighth left rib, posterior aspect of seventh right rib, posterior aspects of forth and sixth left ribs, right scapula, left tibiotalar area, distal diaphysis of left femur, and both of iliac wings (Figure 3). Tru-cut biopsies were performed from skin lesion and mass located in the pancreatic head. Neoplastic cell infiltration intermingled with areas of fibrosis and subtle necrosis, originating in papillary dermis and extending down into subcutaneous tissue corresponding to lower margin of biopsy sample, was seen on skin biopsy (Figure 4). Morphologically, neoplastic cells splayed between collagen bundles in dermis appeared to have plasmacytoid-plasmablastic differentiation. Immunohistochemical staining for CD38 and kappa and lambda light chains was carried out. It revealed that neoplastic cells showed monotypic light chain restriction with positive kappa light chains (Figure 5). Tru-cut biopsy from pancreatic mass was performed and histopathological and immunohistochemical findings were similar to those encountered in skin mass biopsy: neoplastic cell infiltrates interposed between areas of fibrosis and subtle necrosis, plasmacytoid-plasmablastic differentiation, and kappa light chain positive plasma cell dominance (Figures 6 and 7). A final diagnosis of multiple myeloma complicated with extramedullary plasmacytomas involving the pancreas, suprarenal gland, kidney, skin, lung, liver, spleen, and lymph nodes was attained.

We proceeded with chemotherapy. After one cycle of VAD (vincristine, adriamycin, and dexamethasone) and 2 cycles of CHOP (cyclophosphamide, doxorubicin, vincristine, and prednisone) considerable regression of masses has been observed. The patient still continues his treatment.

## 3. Discussion

Plasma cell dyscrasias are a diverse group of disorders which include multiple myeloma, plasma cell leukemia, solitary plasmacytoma of bone, extramedullary plasmacytoma (EMP), Waldenström's macroglobulinemia (WM), primary amyloidosis, light chain deposition disease and heavy-chain disease. Roughly, 30% of the patients suffer from local recurrence and distant metastasis and transformation to multiple myeloma occasionally occur [5]. EMP is a rarely seen type of plasma cell dyscrasias. It constitutes 3-4% of all cases [6] and can be divided into two categories: primary and secondary. The diagnosis requires not only the demonstration of extraosseous myelomatous mass but also excluding bone marrow involvement and demonstration of no involvement in bone scintigraphy [6]. The vast majority of EMPs develop and presents as a mass secondary to multiple myeloma [7]. Multiple myeloma with extraosseous involvement is frequently associated with anaplastic to “undifferentiated” morphology and dictates poor prognosis. A review of more than 400 published articles showed 82.2% of EMPs arose in the upper aerodigestive tract while 17.8% were found in the gastrointestinal tract, urogenital tract, skin, lung, and breast [8]. Liver, spleen, and lymph nodes are common sites of EMPs. We confirmed the presence of EMPs, which were associated with multiple myeloma, in the pancreas, suprarenal gland, kidney, skin, lung, liver, spleen, lymph nodes, and bone. Involvement of pancreas and suprarenal glands in addition to lung, skin, and kidney is extremely rare.

In a database of 2584 patients with multiple myeloma, liver involvement as mass or macroscopic nodule was detected only in 9 patients [9]. Hepatic infiltration related to MM may present as hepatomegaly, jaundice, ascites, and fulminant liver failure. Hepatomegaly associated with plasma cell infiltration was present in 70% of patients while 50–70% of patients had mildly elevated liver transaminase levels [10]. Isolated alkaline phosphatase elevation may be encountered in some patients with hepatic plasma infiltration [11]. Jaundice was the most prominent clinical manifestation and dominated the clinical picture as a result of presence of plasmacytoma in the pancreatic head as well as presence of liver involvement in our patient.

EMPs usually arise in the aerodigestive tract and reticuloendothelial system while pancreatic involvement is exceedingly rare. On CT examination, EMP of the pancreas showed itself as a homogeneous solid mass when compared to the pancreatic adenocarcinoma which was revealed as an irregular and low-density mass [6]. It is a diagnostic challenge facing clinicians to differentiate between EMPs and other lesions seen as homogeneous masses (pancreatic endocrine cell tumors, acinar cell carcinoma), primary and secondary pancreatic malignancies, lymphoma, autoimmune pancreatitis, and clinical scenarios associated with pancreatic inflammation [6, 7, 12]. Plasma cell tumors are very sensitive to radiotherapy. Henceforth, radiation therapy is treatment of choice for patients with localized EMP of the pancreas whereas distal pancreatectomy for lesions in the tail of the pancreas stands out as an available, viable option. In case of multiple myeloma involving the pancreas, systemic chemotherapy seems to be more appropriate. According to treatment data of 18 patients with pancreatic EMPs, four patients received chemotherapy, four radiotherapy, and two chemoradiotherapy and only one patient underwent surgical resection [6].

Primary EMP in the adrenal glands is extremely rare phenomenon. Our case presentation depicts EMP involving the left adrenal gland. Li et al. reported a case with functional disturbances whilst the remaining EMPs in the adrenal glands seemed to be nonfunctional [5]. They are visualized as a mass with smooth borders and heterogeneous hyperintensity on CT and MRI modalities. However, it still remains a challenge for clinician to differentiate EMPs from primary and secondary malignancies of adrenal glands.

Hedinger reported first the relation of cutaneous plasmacytomas to MM, which is quite uncommon, in 1911. There is no precise data available related to the incidence. Plasma cell infiltration is the cause of specific lesions while nonspecific lesions develop owing to accumulation of abnormal proteins (amyloid, cryoglobulins) at the dermoepidermal junction, cytopenia (anemia, thrombocytopenia, and leukopenia), and infiltration of other organs. Crystal deposition may emerge as the first manifestation in some cases. Plasmacytomas developing in association with MM are grouped under the heading of “secondary cutaneous plasmacytomas.” The underlying mechanism of cutaneous infiltration is not fully elucidated so far. However, interleukin-6 is thought to play a part in plasma cell expansion.

Patients with aggressive form tend to be younger, with a mean age of 50, while patients with MM, in general, have an average age of sixty [13]. Pulmonary thromboembolism can present as an indirect manifestation of thoracic involvement. To confirm diagnosis of extramedullary thoracic plasmacytoma is distinctly difficult in case of neither thoracic vertebral nor rib involvement as radiologic findings on CT and MRI are not specific enough to differentiate between EMPs and primary or metastatic carcinomas, sarcoma, neuroendocrine or neuroectodermal tumors, and lymphomas. Consequently, transthoracic needle biopsy and/or sampling through thoracotomy are frequently necessary. Immunohistochemical staining is imperative for precise diagnosis.

Surgical resection or radiotherapy is treatment of choice for primary EMP confined to a single organ. Systemic chemotherapy and stem cell transplantation are recommended for relapsed/refractory disease or multiple plasmacytomas. In our case, we proceeded with systemic chemotherapy due to the fact that the patients relapsed as multiple plasmacytomas arising in various organs following first-line chemotherapy [1–17].

## References

[1] Yoon Y. H., Cho W. I., Seo S. J. (2006). Case of multiple myeloma associated with extramedullary cutaneous plasmacytoma and pyoderma gangrenosum. *International Journal of Dermatology*.

[2] Birjawi G. A., Jalbout R., Musallam K., Tawil A., Taher A., Khoury N. (2008). Abdominal manifestations of multiple myeloma: a retrospective radiologic overview. *Clinical Lymphoma and Myeloma*.

[3] Pehlivan Y., Sevinc A., Sari I. (2006). An interesting cause of esophageal ulcer etiology: multiple myeloma of IgG kappa subtype. *World Journal of Gastroenterology*.

[4] Heß T., Egerer G., Kasper B., Rasul K. I., Goldschmidt H., Kauffmann G. W. (2006). Atypical manifestations of multiple myeloma: radiological appearance. *European Journal of Radiology*.

[5] Li Y., Guo Y.-K., Yang Z.-G., Ma E.-S., Min P.-Q. (2007). Extramedullary plasmacytoma involving the bilateral adrenal glands on MR imaging. *Korean Journal of Radiology*.

[6] Hirata S., Yamaguchi K., Bandai S., Izumo A., Chijiiwa K., Tanaka M. (2002). Secondary extramedullary plasmacytoma involving the pancreas. *Journal of Hepato-Biliary-Pancreatic Surgery*.

[7] Choi E. J., Kim K. A., Park C. M., Lee J. H., Choi J. W., Seol H. Y. (2003). Extramedullary plasmacytoma of the pancreas: imaging findings: case report. *Journal of the Korean Radiological Society*.

[8] Ooi G. C., Chim J. C.-S., Au W.-Y., Khong P.-L. (2006). Radiologic manifestations of primary solitary extramedullary and multiple solitary plasmacytomas. *The American Journal of Roentgenology*.

[9] Wu X.-N., Zhao X.-Y., Jia J.-D. (2009). Nodular liver lesions involving multiple myeloma: a case report and literature review. *World Journal of Gastroenterology*.

[10] Yoon Y. S., Min Y. H., Chon C. Y. (1993). Liver involvement in multiple myeloma proven by peritoneoscopy—a case report. *Yonsei Medical Journal*.

[11] Solves P., De La Rubia J., Jarque I. (1999). Liver disease as primary manifestation of multiple myeloma in a young man. *Leukemia Research*.

[12] Hiller N., Goitein O., Ashkenazi Y. J. (2004). Plasmacytoma of the pancreas. *The Israel Medical Association Journal*.

[13] Cabrera A., Klein J. S. (1997). Bilateral pleural masses and shortness of breath associated with multiple myeloma. *Chest*.

[14] Shirdel A., Attaran D., Ghobadi H., Ghiasi T. (2007). Myelomatous pleural effusion. *Tanaffos*.

[15] Oshima K., Kanda Y., Nannya Y. (2001). Clinical and pathologic findings in 52 consecutively autopsied cases with multiple myeloma. *The American Journal of Hematology*.

[16] Güvenç B., Canataroğlu A., Gümürdülü Y., Gümürdülü D., Paydaşt S. (2001). Multiple myeloma with skin involvement. *Journal of the European Academy of Dermatology and Venereology*.

[17] del Giglio A., Weinschenker P., de Araújo Burgos Manhani A. R., Ippolito Carbonell A. L., Mitteldorf C. A. T. S. (2005). Hepatic plasmacytosis as a manifestation of relapse in multiple myeloma treated with thalidomide. *Southern Medical Journal*.

